# Research Progress on Plant Long Non-Coding RNA

**DOI:** 10.3390/plants9040408

**Published:** 2020-03-25

**Authors:** Ling Wu, Sian Liu, Haoran Qi, Heng Cai, Meng Xu

**Affiliations:** 1 Co-Innovation Center for Sustainable Forestry in Southern China, Key Laboratory of Forest Genetics and Biotechnology of Ministry of Education, Nanjing Forestry University, Nanjing 210037, China; lingwuwl@foxmail.com (L.W.); sianliu@yzu.edu.cn (S.L.); hengcai@njfu.edu.cn (H.C.); 2 College of Horticulture and Plant Protection, Yangzhou University, Yangzhou 225009, China

**Keywords:** non-coding RNA, epigenetic modification, strand-specific library, CRISPR/Cas9 system

## Abstract

Non-coding RNAs (ncRNAs) that were once considered “dark matter” or “transcriptional noise” in genomes are research hotspots in the field of epigenetics. The most well-known microRNAs (miRNAs) are a class of short non-coding, small molecular weight RNAs with lengths of 20–24 nucleotides that are highly conserved throughout evolution. Through complementary pairing with the bases of target sites, target gene transcripts are cleaved and degraded, or translation is inhibited, thus regulating the growth and development of organisms. Unlike miRNAs, which have been studied thoroughly, long non-coding RNAs (lncRNAs) are a group of poorly conserved RNA molecules with a sequence length of more than 200 nucleotides and no protein encoding capability; they interact with large molecules, such as DNA, RNA, and proteins, and regulate protein modification, chromatin remodeling, protein functional activity, and RNA metabolism in vivo through *cis*- or *trans*-activation at the transcriptional, post-transcriptional, and epigenetic levels. Research on plant lncRNAs is just beginning and has gradually emerged in the field of plant molecular biology. Currently, some studies have revealed that lncRNAs are extensively involved in plant growth and development and stress response processes by mediating the transmission and expression of genetic information. This paper systematically introduces lncRNA and its regulatory mechanisms, reviews the current status and progress of lncRNA research in plants, summarizes the main techniques and strategies of lncRNA research in recent years, and discusses existing problems and prospects, in order to provide ideas for further exploration and verification of the specific evolution of plant lncRNAs and their biological functions.

Non-coding RNAs (ncRNAs) constitute a group of genetic information-containing molecules generated from in vivo genome transcription, but they do not encode proteins. Once considered as “dark matter” or “transcriptional noise” in the genome [1], lncRNA has become one of the main topics of epigenetic research [2]. Whole-genome and transcriptome sequencing results have shown that more than 75% of the human genome, and more than 50% of the *Arabidopsis thaliana* genome could be transcribed into RNA. Among these transcripts, protein-coding sequences only account for approximately 1.5%, and most are ncRNAs that lack a protein translation function but play important roles in the growth and development of eukaryotes [3]. According to their functions, ncRNAs can be divided into constitutive ncRNAs and regulatory ncRNAs [4]. The former includes ribosomal RNA (rRNA), transfer RNA (tRNA), small nuclear RNA (snRNA), and small nucleolar RNA (snoRNA); the latter mainly includes short interfering RNA (siRNA), miRNA, Piwi-interacting RNA (piRNA), circular RNA (circRNA) and long ncRNA (lncRNA) [5]. 

## 1. The Source of lncRNA and Regulatory Mechanisms

### 1.1. Definition and Source of lncRNA

lncRNAs are a class of transcripts with low sequence conservation in different species, a length of over 200 nucleotides, and no protein-coding ability [6]. Most lncRNAs are transcribed by RNA polymerase II; however, some lncRNAs are transcribed by RNA polymerase III, and a small number of lncRNAs in plants are produced by plant-specific RNA polymerases IV and V. Most lncRNAs have specific spatial structures and spatiotemporal expression patterns. According to the position of lncRNAs relative to adjacent protein-coding genes in the genome, lncRNAs can be divided into five types: sense lncRNA, antisense lncRNA, bidirectional lncRNA, intronic lncRNA (incRNA), and large intergenic lncRNA (lincRNA). Sense or antisense lncRNA refers to the overlapping of these lncRNAs with one or more exons of a protein-coding gene in the same strand or complementary strand; bidirectional lncRNA emerges close to the transcription start site of coding genes adjacent to the complementary strand but run in the opposite direction; intronic lncRNA originates from introns of protein-coding genes, and incRNA is generated from the intergenic regions of two protein-coding genes [4,7].

Previous studies have shown that lncRNA may originate from several pathways: (A) the coding frame is inserted between the introns of a protein-coding gene, and the inserted coding frame is recombined with the previous coding sequence to form a functional lncRNA; a typical representative of this pathway is lncRNA *Xist*, which can induce the inactivation of the mammalian X chromosome; (B) after chromosomal rearrangement, two separated non-transcribed regions are joined together to form an ncRNA with multiple exons; (C) ncRNAs are replicated through retrotransposition to form functional ncRNAs or non-functional pseudogenes; (D) ncRNAs containing adjacent repeats are generated from tandem repeats; and (E) the formation of functional ncRNAs is caused by the intersection of transposons [4].

### 1.2. The Regulatory Mechanisms of lncRNA

lncRNAs are not only abundant in eukaryotes but also have various biological functions, and their action mechanisms are complex and diverse. lncRNAs can interact with macromolecules to regulate protein modification, chromatin remodeling, protein functional activity, and RNA metabolism at multiple levels (including transcriptional, post-transcriptional, and epigenetic levels). In addition, the transcription, splicing patterns, and secondary structure of lncRNAs are also regulated by RNA modification, DNA methylation, and histone modification [8,9]. For miRNAs, the target gene can be determined by complementary pairing with the target sequence [10,11]; however, the regulatory mechanisms of lncRNAs are complex and diverse, and a target gene may be regulated by both *cis*-action (short-range) and *trans*-action (long-distance). Wang et al. [12] classified various functions of lncRNAs into four regulatory mechanisms. 

(1) Signal—These lncRNAs can act as signaling molecules to bind transcription factors or participate in signaling pathways to further regulate the spatiotemporal expression of protein-coding genes. 

(2) Decoy—These lncRNAs can only act as decoys to indirectly regulate the expression levels of protein-coding genes through the recruitment of RNA-binding proteins (e.g., transcription factors, chromosome modifications, and regulatory molecules). lncRNA *Gas5* acts as a decoy to prevent the binding of the corticosteroid receptor to chromosomes [13]. In addition, these lncRNAs can also act as “sponges” to competitively adsorb complementary miRNAs to indirectly regulate the functions of miRNA target genes; therefore, these lncRNAs are also known as competitive endogenous RNAs (ceRNAs) [14]. In fact, the principle of this phenomenon had been identified a while ago in plants, where it is known as ‘‘target mimicry’’. [15,16] In Arabidopsis, miR399 and its target gene, *PHOSPHATE 2* (*PHO2*), are known to play a role in the maintenance of phosphate homeostasis [17]. The lncRNA, *INDUCED BY PHOSPHATE STARVATION1* (*IPS1*) and *At4,* both competitively bind to miR399 and upregulate the *PHO2* expression levels [18,19]. 

(3) Guide—As guides of RNA-binding proteins, these lncRNAs guide ribonucleoprotein complexes to specific locations or recruit chromatin-modifying enzymes to target genes. For genes that are close to lncRNAs, regulation occurs through *cis*-action, and for genes that are far away from lncRNAs, regulation occurs through *trans*-action, thereby changing the expression levels of target genes. For example, lncRNA *Enod40* in *Medicago truncatula* can directly bind with RNA-binding proteins to participate in the formation of root nodules [20]. Two Arabidopsis lncRNAs, *COOLAIR* and *COLDAIR,* recruit the protein complex Polycomb Repressive Complex 2 (PRC2) to the *FLOWERING LOCUS C* (*FLC*) locus, and PRC2 promotes H3K27 methyltransferase activity to change the chromatin structure of the *FLC* locus, thereby inhibiting the expression of *FLC* and regulating flowering time [21]. 

(4) Scaffold—Traditionally, proteins have been used as scaffolds in many complexes, but recent studies have shown that lncRNAs can also act as scaffolds to combine multiple proteins together to form ribonucleoprotein complexes. These lncRNAs often have a binding domain that can bind proteins and other regulatory molecules, and transcriptional activation or inhibition can occur at the same time and in the same space. Plant-specific RNA polymerase Pol V can produce cytoskeleton-related transcripts required for siRNAs and associated proteins, and allow chromatin modification at specific sites of target genes [8]. 

In addition to the four regulatory mechanisms, lncRNAs can also serve as precursors for the synthesis of short ncRNAs (siRNAs and miRNAs), which has been confirmed in a variety of plants [22]. Furthermore, Ponting et al. [2] divided the roles of lncRNAs in transcription regulation into nine categories. Unfortunately, many lncRNA regulatory mechanisms are mostly derived from animals. So far, only a few lncRNA mechanisms in plants have been revealed, resulting in a lack of systematic and consensus lncRNA regulatory mechanisms in the plant. However, following the deepening of plant lncRNA research, the regulatory mechanism of lncRNA in the plant will be more perfect.

## 2. The Extensive Involvement of lncRNAs in Plant Growth and Development and Stress Response Processes

Research on plant lncRNAs is just beginning, and it has gradually emerged in the molecular biological studies of *Arabidopsis*, *Oryza sativa*, *Medicago sativa*, *Zea mays*, *Solanum lycopersicum*, *Gossypium* ssp. (*Gossypium barbadense* and *Gossypium hirsutum*), and other herbaceous model plants (Table 1). The star molecules *COLDAIR* and *COOLAIR* are two lncRNAs generated in the first intron and the antisense strand, respectively, of *FLC*, the flowering repressor of *A. thaliana*. The former has a 5‘-end cap structure but lacks a 3’-end polyA tail. In the process of spring flowering, *COLDAIR* recruits the protein complex PRC2 to activate H3K27me3 to maintain the low-level expression of the *FLC* gene [21]. The latter involves alternative splicing (AS) with a typical 5‘-end cap structure and a 3’-end polyA tail and can indirectly inhibit *FLC* expression through transcription interference [23]. The Arabidopsis *ELF18-INDUCED LONG-NONCODING RNA1* (*ELENA1*) promotes *PATHOGENESIS-RELATED GENE1* (*PR1*) expression through dissociating the *FIBRILLARIN 2* /*Mediator subunit 19a* (*FIB2*/*MED19a*) complex and releasing *FIB2* from PR1 promoter [24,25]. The lncRNA *MAS* (one NAT-lncRNA, *NAT-lncRNA_2962*) transcribed on the antisense strand of the floral inhibitor *MADS AFFECTING FLOWERING4* (*MAF4*) is induced by cold treatment and can inhibit premature flowering in *A. thaliana* through the activation of *MAF4* expression by the COMPA-like complex involved in histone H3K4me3 modification [2]. The lncRNA *AUXIN-REGULATED PROMOTER LOOP* (*APOLO*) is transcriptionally regulated by auxin and modulates later root development through mediating the formation of a chromatin loop (R-loop) encompassing the promoter of its neighboring gene *PID* and suppressing the *PID* transcript [26,27,28]. Single base mutations in *LDMAR* in photoperiod-sensitive male sterility (PSMS) in rice can change the secondary structure of *LDMAR*, causing an increase in the DNA methylation level in the promoter region of *LDMAR* to suppress its transcription under long-day treatment [29]; another lncRNA, *PMS1T*, regulates PSMS by phased siRNAs generated from miR2118 targeted splicing [30,31].

For lncRNAs in higher plants, the earliest literature reports that lncRNA *Enod40* in *M. truncatula* can produce biological functions through the production of short peptides during the process of root nodule formation [32], and that *ASCO*-lncRNA generated by the transcription of *ENOD40* homologs in *A. thaliana* can compete with messenger RNA (mRNA) to bind nuclear speckle RNA-binding protein (NSR), AS regulators, thus affecting AS patterns and expression levels of downstream auxin response genes and further regulating root development in *A. thaliana* [33]. Additionally, an increasing number of lncRNAs involved in plant growth, development, and stress response processes and their biological functions are being revealed. The results show that the lncRNA *HIDDEN TREASURE 1* (*HID1*) modulated by continuous red light also transcriptionally represses *PHYTOCHROME-INTERACTING FACTOR 3* (*PIF3*) and promotes seedling photomorphogenesis in Arabidopsis [34], the *antisense heat stress transcription factor2a* (*asHSFB2a)* overexpression in Arabidopsis results in the loss of *HSFB2a*, which impairs the development of female gametophytes [35], *Brassica campestris Male Fertility11* (*BcMF11)* plays an essential role in pollen development and male fertility [36,37], the overexpression of *Leucine-rich repeat receptor kinase antisense intergenic RNA* (*LAIR*) can increase rice yield [38], the inhibition of *TWISTED LEAF* (*TL*) expression can result in helical twisting of rice leaves [39]. Furthermore, *DROUGHT INDUCED lncRNA* (*DRIR*) and *SVALKA* participate in the stress response processes in *A. thaliana* [40,41], and lncRNAs such as *GhlncNAT-ANX2*, *GhlncNAT-RLP7*, *lncRNA16397,* and *lncRNA33732* play important roles in cotton and tomato resistance to pathogen infection [42,43,44]. *IPS1* in *A. thaliana* and *PILNCR1* in maize, induced by low phosphorus stress, contain miRNA response elements and can act as ceRNAs to bind with miR399 without being cleaved by miR399, thus protecting miR399-target genes and participating in the regulation of phosphate homeostasis in plants [45]. *AtR8*, which is transcribed from RNA polymerase III, is specifically expressed in the roots of *A. thaliana* and is involved in the response to hypoxia stress [46]. The apple lncRNA, *MSTRG.85814.11*, acts as a transcriptional enhancer of *SAUR32* and contributes to the Fe deficiency response [47].

Studies targeting herbaceous model plants have revealed the biological functions and mechanisms of a small number of lncRNAs. It has been gradually recognized that lncRNAs widely mediate the transmission and expression of genetic information, thereby regulating plant growth and development, secondary metabolism and stress adaptation. The growth and stress response mechanisms of trees are more complex than those of herbaceous plants. In recent years, lncRNAs in trees have also emerged based on high-throughput sequencing and biological information prediction [48,49], and lncRNAs have been found in various stages of tree growth and stress response processes [10,48,50]. The authenticity identification and annotation of these lncRNAs have been enormously challenging, and there is still a long way to go for further experimental verification and validation. To date, there have been no reports on the biological functions and mechanisms of lncRNAs in trees.

## 3. Research Strategies Regarding Plant lncRNAs 

### 3.1. Screening and Identification of lncRNAs Based on High-Throughput Sequencing

Transcriptome library construction and sequencing cannot separate the sense strand and the antisense strand, making it difficult to identify and screen a large number of lncRNAs. With the wide application of a strand-specific library, the continuous development of lncRNA prediction algorithms, and the continuous updating of plant genome sketches, a large number of potential lncRNAs have been screened and identified in plants such as *Arabidopsis* [2], *O. sativa* [54], *Brassica oleracea* [55], and cotton [56]. Based on the development of third-generation sequencing technology, information regarding lncRNAs on the sense strand and the antisense strand can be obtained by full-length transcriptome sequencing without the construction of a strand-specific library. Currently, a large number of lncRNAs have been identified and screened by PacBio sequencing in *Arabidopsis* [57], *O. sativa* [58], maize [59], and other species [60,61,62,63,64,65]. These results show that compared with the ssRNA-seq, Pacbio sequencing can obtain longer lncRNA.

### 3.2. lncRNA Expression and Localization Techniques

The abundance of lncRNA expression in plants is low, generally only 1/30 to 1/60 of the average mRNA expression [66]. For the detection and localization of the expression levels of a large number of potential lncRNAs, Northern blot, RNA fluorescence in situ hybridization (RNA FISH), real-time quantitative polymerase chain reaction (qRT-PCR) and transient expression are generally used. qRT-PCR has the advantages of high sensitivity, high specificity, and ease of operation; therefore, it is the first choice for the validation of high-throughput transcriptome expression and the detection of low-enriched lncRNA expression. Transient expression in protoplasts and RNA FISH allows exploring the subcellular localization of lncRNAs. Based on a tobacco transient transformation system, Kinoshita et al. [67] found *nonprotein conding 60* (*NPC60*) is a nuclear-localized lncRNA with a length of approximately 500 nt, which involved in *Arabidopsis* stress responses [26]. Qin et al. [40] detected lncRNA *DRIR* localized in the nucleus using RNA FISH. Yang et al. [68] detected strong fluorescence signals of lncRNA*^CCS52B^* in peduncles during early flower development using RNA FISH; however, this lncRNA was not expressed in the shoot apical meristem and floral primordium. The plant protoplast contains all the parts of a cell without a cell wall and is a complete “naked cell” with physiological, biochemical, and metabolic activities. Plant protoplasts provide a multifunctional cell-based experimental platform. The greatest advantage of transient expression in protoplasts is rapid detection and high throughput. This technique has been applied in studies on the regulation of lncRNA, target gene expression, subcellular localization, signal transduction, and protein interactions [10,50].

### 3.3. Strategies Based on Acquisition/Loss of Function

Molecular detection and phenotypic comparison analysis of transgenic plants with stable expression through genetic transformation are the "gold standards" for gene function verification. Based on large-scale identification and validation in protoplast transient expression systems, the biological functions of candidate lncRNAs can be further revealed through highly efficient and stable plant genetic transformation systems. The basic strategy for the functional verification of plant lncRNAs is still a strategy based on acquisition/loss of function, i.e., studies on lncRNA overexpression based on genetic transformation platforms or loss-of-function studies based on clustered regularly interspaced short palindromic repeat (CRISPR)-associated protein 9 (Cas9) systems, RNAi and siRNA. Yu et al. [52] obtained lncRNA *ALEX1* (a full-length of 294 nt) through the rapid amplification of cDNA ends (RACE) technology, and the construction of an overexpression vector and genetic transformation revealed that *ALEX1* could activate the jasmonic acid signaling pathway and affect resistance to bacterial blight in rice. Cui et al. [43] analyzed the overexpression and silencing of *lncRNA33732* in tomatoes and found that *lncRNA33732* can enhance the resistance of tomatoes to Phytophthora infestans by inducing the expression of respiratory oxidase homolog (RBOH) and the accumulation of H_2_O_2_. Li et al. [53] used CRISPR/Cas9 gene-editing technology to obtain loss-of-function mutants of *lncRNA1459*. Compared with wild-type tomato, the tomato ripening process was significantly repressed in *lncRNA1459* mutants, ethylene production, and lycopene accumulation were largely repressed in *lncRNA1459* mutants, and the expression levels of numerous ripening-related genes and lncRNAs changed significantly. Liu et al. [39] found an endogenous lncRNA, *TL*, in rice, and TL-RNAi transgenic lines were obtained through RNAi technology. For the *TL*-RNAi transgenic lines, the leaves were distorted, and the expression of *OsMYB60* was significantly increased, suggesting that *TL* may play a cis-regulatory role in *OsMYB60* expression during leaf morphological development.

### 3.4. Epigenetic Regulation of lncRNA

lncRNAs play important roles in epigenetic modification processes, such as histone modification, DNA methylation, and chromosomal remodeling [66]. They can affect the expression levels of target genes by changing the chromatin or DNA modification states of target genes, which is achieved by binding and recruiting specific epigenetic enzyme complexes to target gene regions. Some lncRNAs may interact with proteins; therefore, RNA-pull down technology or RNA immunoprecipitation (RIP-seq) can be used to investigate interactions between candidate lncRNAs and proteins. Some candidate lncRNAs may play roles in the epigenetic modification; therefore, chromatin immunoprecipitation (ChIP-seq) can be used to investigate the roles of these lncRNAs in methylation modifications. Based on ChIP, ChIP-qPCR, RIP, and other experiments, several lncRNAs mentioned above, including *COOLAIR*, *COLDAIR*, *APOLO*, *ELENA*, *DRIR*, *SVALKA*, *ASCO*-lncRNA, *MAS* and *LDMAR*, are all classically regulated through epigenetic regulation. Moreover, Wu et al. [69] found that the methylation level of H3K27me3 in Arabidopsis *AG-incRNA4* RNAi was significantly lower than that of the control by performing ChIP-seq. The RNA-pulldown and RIP techniques were used to further reveal that the sense strand of *AG-incRNA4* can directly bind to the CURLY LEAF (CLF) protein. Wang et al. [70], using ChIP-seq, showed that the lncRNA *At4* was a direct target of *general control non-repressed protein 5* (*GCN5*) under the stress of phosphate starvation. Liu et al. [51] studied the binding between NLP7 and NRE-like elements in the promoter of lncRNA *T5120*. Using ChIP-qPCR, they found that only in the presence of nitrate can NLP7 bind to NRE-like elements in the promoter of lncRNA *T5120*. 

## 4. Prospects

There is no doubt that compared with the abundant “dark matter” of the genome of higher plants, research progress regarding lncRNAs in herbaceous model plants has only begun. The sequence similarity between plant lncRNAs is weak, and the action mechanisms are complex and diverse. The action modes of lncRNAs revealed in model plants are difficult to apply to other plants [10,50]. It is difficult to comprehensively identify the authenticity and biological functions of plant lncRNAs through lncRNA overexpression (i.e., function acquisition studies); more efficient and specific targeted gene-editing technology is the inevitable choice for plant lncRNA research. Compared with traditional gene-editing technologies, such as zinc finger nuclease (ZFN) and transcription activator-like effector nuclease (TALEN), CRISPR/Cas9 gene-editing technology has been widely used due to its advantages of simplicity, high efficiency, and strong expandability. It has been used in the genetic engineering of multiple plants, including A. thaliana, rice, and poplar. It is reasonable to believe that with continuous improvements in the use of CRISPR/Cas9 technology for studying important growth and development traits of plants [71,72], multilevel exploration and verification of species-specific lncRNAs and their biological functions will not only enrich and expand the theoretical basis of plant organogenesis and morphogenesis but also provide different scientific perspectives for enhancing and improving the adaptability of plants to stress.

## Figures and Tables

**Table 1:** Summary of functionally validated lncRNAs in plants.

LncRNA Name	Species	Biological Functions	Refs
*PHO2*	*Arabidopsis thaliana*	Phosphate homeostasis	[17]
*COLDAIR*	*Arabidopsis thaliana*	Vernalization response	[21]
*COOLAIR*	*Arabidopsis thaliana*	Vernalization response	[23]
*ELENA1*	*Arabidopsis thaliana*	Immune response	[24,25]
*MAS*	*Arabidopsis thaliana*	Vernalization response	[2]
*APOLO*	*Arabidopsis thaliana*	Later root development	[26,27,28]
*ASCO*	*Arabidopsis thaliana*	Root development	[33]
*HID1*	*Arabidopsis thaliana*	Seedling photomorphogenesis	[34]
*asHSFB2a*	*Arabidopsis thaliana*	Gametophyte development, heat stress response	[35]
*DRIR*	*Arabidopsis thaliana*	Drought and salt stress responses	[40]
*SVALKA*	*Arabidopsis thaliana*	Cold stress response	[41]
*IPS1*	*Arabidopsis thaliana*	Phosphate homeostasis	[45]
*AtR8*	*Arabidopsis thaliana*	Hypoxia stress response	[46]
*NPC60*	*Arabidopsis thaliana*	Stress responses	[26]
*T5120*	*Arabidopsis thaliana*	Nitrate response	[51]
*LDMAR*	*Oryza sativa*	Photoperiod-sensitive male sterility	[29]
*PMS1T*	*Oryza sativa*	Photoperiod-sensitive male sterility	[30,31]
*LAIR*	*Oryza sativa*	Rice yield	[38]
**TL**	*Oryza sativa*	Leaf morphological development	[39]
*ALEX1*	*Oryza sativa*	Disease resistance	[52]
*lncRNA16397* *lncRNA33732*	*Solanum lycopersicum*	Pathogen infection	[42,43]
*lncRNA1459*	*Solanum lycopersicum*	Tomato ripening process	[53]
*PILNCR1*	*Zea mays*	Phosphate homeostasis	[45]
*Enod40*	*Medicago truncatula*	The formation of root nodules	[20]
*BcMF11*	*Brassica campestris* ssp. *chinensis*	Pollen development, male fertility	[36,37]
*GhlncNAT-ANX2GhlncNAT-RLP7*	*Gossypium* ssp.	Pathogen infection	[44]
*MSTRG.85814.11*	*Malus domestica*	Fe deficiency response	[47]
